# A Possible Anticancer Agent, Type III Interferon, Activates Cell Death Pathways and Produces Antitumor Effects

**DOI:** 10.1155/2011/479013

**Published:** 2011-10-16

**Authors:** Masatoshi Tagawa, Kiyoko Kawamura, Quanhai Li, Yuji Tada, Kenzo Hiroshima, Hideaki Shimada

**Affiliations:** ^1^Division of Pathology and Cell Therapy, Chiba Cancer Center Research Institute, 666-2 Nitona, Chuo-ku, Chiba 260-8717, Japan; ^2^Department of Molecular Biology and Oncology, Graduate School of Medicine, Chiba University, 1-8-1 Inohana, Chuo-ku, Chiba 260-8670, Japan; ^3^Department of Respirology, Graduate School of Medicine, Chiba University, 1-8-1 Inohana, Chuo-ku, Chiba 260-8670, Japan; ^4^Department of Pathology, Tokyo Women's Medical University Yachiyo Medical Center, 477-96 Owada-Shinden, Yachiyo 276-8524, Japan; ^5^Department of Surgery, School of Medicine, Toho University, Tokyo 143-8540, Japan

## Abstract

Recently identified interleukin-28 and -29 belong to a novel type III interferon (IFN) family, which could have distinct biological properties from type I and II IFNs. Type I IFNs, IFN-**α**/**β**, have been clinically applied for treating a certain kind of malignancies for over 30 years, but a wide range of the adverse effects hampered the further clinical applications. Type III IFNs, IFN-**λ**s, have similar signaling pathways as IFN-**α**/**β** and inhibits proliferation of tumor cells through cell cycle arrest or apoptosis. Restricted patterns of type III IFN receptor expression in contrast to ubiquitously expressed IFN-**α**/**β** receptors suggest that type III IFNs have limited cytotoxicity to normal cells and can be a possible anticancer agent. In this paper, we summarize the current knowledge on the IFN-**λ**s-mediated tumor cell death and discuss the functional difference between type I and III IFNs.

## 1. Introduction

Interferons (IFNs) have been described as agents mediating antiviral responses over the years; however, further investigations are still required to clarify the biological properties and the mechanisms responsible for the functions [1]. There are 3 IFN families currently known, which have different receptor structures [2, 3]. Type I IFN family consists of IFN-*α* and IFN-*β* in human and binds a common heterodimeric receptor complex, composed of IFNAR1 and IFNAR2 [4]. Type II IFN comprises of IFN-*γ*, which is not homologous to type I IFNs in the structure, and binds a different receptor complex, IFNGR1 and IFNGR2 [2, 3]. The interaction of type I IFNs with the receptor complex induces activation of signal transducer and activator of transcription (STAT) family members, resulting in complex formations with different transcription factors [4]. IFN-*γ* shares similar but distinct signal transduction pathways compared with that of type I IFNs and has biologically different functions from type I IFNs.

Type III IFNs, the newest IFN family, has been identified as IFN-*λ* which includes 3 subtypes, IFN-*λ*1, -*λ*2 and -*λ*3, also known as interleukin-29 (IL-29), IL-28A, and IL-28B, respectively, [5, 6]. The receptor complexes have also been identified, and the interaction between the ligand and the receptors seems to activate identical signal transduction pathways as do type I IFNs. Nevertheless, type III IFNs could have different functions from the type I IFNs since type III IFNs bind a specific receptor complex with restricted expression manners.

## 2. Structure of Type III IFNs and the Receptor Complex

Type III IFNs are similar to IL-10 family cytokines in the structure as well as the type I IFNs [5, 6]. Type III IFNs can thereby represent a possible evolutionary linkage between the type I IFNs and the IL-10 family. All the *type III IFN* genes are clustered on chromosome 19 in human and consist of several exons, whereas the *type I IFNs* are mapped on chromosome 9 with a single exon. Murine *type III IFN* genes have also been indentified, *mIFN-*λ*1, -*λ*2 and -*λ*3,* but the *mIFN-*λ*1* gene has a stop codon, producing nonfunctional truncated protein [7]. Interestingly, sequences of bird and zebra fish *IFNs* suggest that type III IFNs may represent an ancestral IFN prototype that gave rise to intron-less type I IFNs by retroposition events and gene duplications [8–10].

All of the type III IFNs bind the same heterodimeric receptor, consisting of a newly identified subunit, IL-28R*α*, and the IL-10R*β* subunit. IL-10R*β* is a subunit of the receptor complex for IL-10 and the IL-10-related cytokines such as IL-22 and IL-26 [11]. Similar to other class II cytokines receptors, IL-28R*α* seems to determine the ligand binding specificity and recruit intracellular signaling molecules. IL-28R*α* is also alternatively spliced to produce 2 variants receptors; one encodes a receptor with a 29-amino acids deletion in the intracytoplasmic portion and the other only encodes the ectodomain. Biological significances of the isoforms remain uncharacterized, but they could serve as a dominant negative form to inhibit the ligand binding or the signal transduction.

The type I IFN receptors are expressed in virtually all the somatic cells. In contrast, IL-28R*α* expression seems to be restricted in a tissues-specific manner although IL-10R is ubiquitously expressed. The IL-28R*α* transcripts are undetectable in several cell types such as fibroblastic and endothelial cells [12]. The limited expression of IL-28R*α* is also shown in tumor cells, and the restricted expression of the receptor complex determines the repertoire of type III IFNs responsiveness, which may generate distinct biological functions from type I IFNs. The IL-28R*α* expression levels are different even among the same cell lineages as found in melanoma cells [13], but it is uncertain that the levels are correlated with the responsibility to type III IFNs. Interestingly, the expression, which was evidenced in the majority of human melanoma specimens, was not identified in premalignant benign nevi specimens [13]. IL-28R*α* can be inducible as type I IFN receptors; peripheral blood mononuclear cells negative for the IL-28R*α* became positive for the expression when treated with IL-4 and granulocyte-macrophage colony-stimulating factor treatments [14].

## 3. Signaling Pathways

Antiviral responses are one of the major functions of IFNs, and the Toll-like receptors- (TLRs-) mediated pathways are essential in sensing of pathogens. The receptors detect most types of viruses by recognizing the nucleic acids and act as prototypical receptors to activate innate immunity. In particular, both TLR8 and TLR9 contribute to type I IFN production. Almost all of the nucleated cells response to viral infection and secrete type I IFNs, in which a number of molecules are involved including retinoic acid inducible gene-I (RIG-1) [15]. The same TLR8 and TLR9 activate type III IFNs production, and the induction mechanisms seem to be similar to those of type I IFNs [16]. Nevertheless, stimulation by either RNA or DNA viruses was less potent to produce type III IFNs compared with type I [17]. In addition, type III IFNs expressions were further augmented by IFN-*α* through their upregulated TLRs- and RIG-I-mediated signaling pathways. In contrast, hepatitis C virus infection induced rather *IFN-*λ*s* but not *IFN-α* or *IFN-*β** mRNA [18]. These data imply that type III IFNs cover the different range of virus infections from type I IFNs and can interact with other cytokines for antiviral activities.

**Figure 1:**
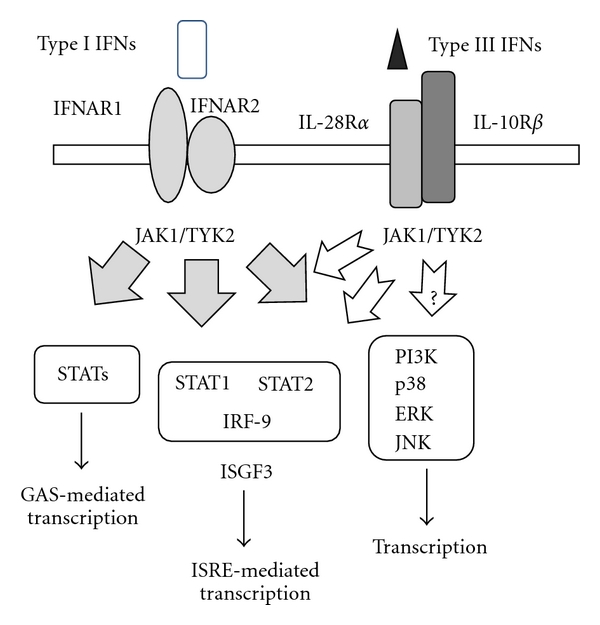
Signaling pathways mediated by type I and type III IFNs. Type I IFNs binding to the receptor complex induces JAK1 and TYK2 activation and phosphorylation of STAT1 and STAT2. The phosphorylated STAT1/STAT2 complex with IRF-9 forms ISGF3, which binds ISRE and initiates a number of transcriptions. Type I IFNs also activate STATs without forming ISGF3 and transactivate IFN-inducible genes through GAS elements. Additionally, type I IFNs activate the PI3K and p38 pathways to stimulate transcription of relevant genes through a number of transcription factors such as AP-1. Similarly, type III IFNs induce the JAK-STATs pathways; however, it is currently unknown whether type III IFNs activate the PI3K and p38 pathways.

Intercellular signal cascade systems are shared between type I and III IFNs (Figure 1). Both type IFNs activate Janus tyrosine kinase- (JAK-) STATs pathways. Ligation of the IFNs with respective receptors results in rapid phosphorylation and activation of the receptor-associated tyrosine kinase 2 (TYK2) and JAK1, which in turn induce phosphorylation and activation of STAT1, STAT2, STAT3, and STAT5. These activated STATs form hetero- or homodimeric structures, which are subsequently translocated to nucleus and bind to IFN-stimulated response elements (ISREs) in regulatory regions of the IFN-stimulated genes (ISGs). ISG factor 3 (ISGF3) is a transcriptional complex, composed of phosphorylated STAT1 and STAT2, and IFN-regulatory factor-9 (IRF-9) and initiates transcriptions of ISGs. Phosphorylated STATs complexes also bind the IFN-*γ* activation site (GAS) and start transcriptions of ISGs. A possible difference between type I and III IFNs could be prolonged activation of STAT1 and STAT2 by type III IFNs, which is accompanied by* de novo* STATs protein synthesis and delayed degradation [19]. The downstream signaling of type III IFNs itself is indistinguishable from that of type I IFNs. A microarray analysis demonstrated IFN-*λ*1 upregulated 60 genes, most of which belong to ISGs group and are the same as found in IFN-*α* stimulation [13]. For example, 2′,5′OAS and myxovirus resistance protein (MxA), both of which are involved in viral protection, are induced by type I and type III IFNs and, likewise, expression levels of major histocompatibility complex (MHC) class I molecules, being favorable for antiviral immunity, are also upregulated in type I and type III IFNs-treated cells. Several lines of studies indicated that the induction levels were lower in type III than in type I IFNs, which may be attributable to a possible difference in the activation processes between the types. These evidences also raise a question as to the biological significance of type III IFNs in host defense mechanisms.

IFN pathways have an alternative circuit besides the JAK-STATs-mediated system. The phosphatidyl-inositol-3-kinase (PI3K) and the p38 kinase pathways have a certain role in the IFN-induced signal transduction. More importantly, activation of the PI3K pathway is dependent on cell types and the p38 kinase pathway can modulate type-I-IFN-dependent responses. It is however currently unknown whether type III IFNs can activate the PI3K and the p38 kinase pathways. A recent study nevertheless showed that type III IFNs induced the activation of the mitogen-activated protein (MAP) and both the p38 and Jun N-terminal kinase-MAP kinases were involved in the gene expression [20]. On the other hand, a different study with human melanoma cells implied that type III IFNs did not activate AKT or extracellular signal-regulated kinases [13] and a possible involvement of the alternative pathways in type III IFNs signaling is thereby controversial.

## 4. Growth Inhibitory Action

IFNs have a growth inhibitory action, which can represent one of the antiviral actions in host defense mechanism due to eliminating virally infected cells. Type I IFNs have been well documented to suppress growth of tumor cells through inducing apoptosis and cell cycle arrest. Procaspases are cleaved by IFN-*α* and IFN-*β* to induce apoptotic cell death, and Takaoka et al. showed that the type I IFNs augmented expression levels of the *p53* tumor suppressor gene, which suggest a close linkage between the antiviral function and the antitumor activity [21]. The p53 induction level by type I IFNs was however relatively low, and we believe that the induced p53 level will not activate apoptotic pathways [22]. Moreover, p53-mutated tumor cells were subjected to IFN-mediated apoptosis [23]; thereby, type-I-IFNs-mediated apoptosis can be rather p53 independent. Type III IFNs also induced apoptosis but it was observed in only some of cell lines derived from the same lineage. For example, esophageal carcinoma cells express the type III IFN receptors, and treatments with type III IFNs upregulated the MHC class I expression and produced antiviral molecules, 2′,5′OAS and MxA, in all the cells. Growth suppression by type III IFNs was thus observed in a third of esophageal carcinoma cells tested [24], suggesting the discrete pathways between the antiproliferative action and the other activities. A repertoire of type-I-IFNs-sensitive cells is the same as that of type III IFNs as far as we tested with the 9 kinds of esophageal carcinoma cells: type-I-IFNs-sensitive cells were also susceptible to type III and vice versa. It is interesting to know whether type III IFNs produce better growth inhibitory actions than type I IFNs. Maher et al. showed that type III IFNs produced greater growth inhibitory effects than IFN-*α* in a human keratinocyte cell line [19]. Direct comparison of the inhibitory ability between type I and type III IFNs is however difficult because the biological action per the IFN protein amount cannot be fairly judged. These data suggest that signal transductions involved in the growth inhibition are distinct from those of other functions such as antiviral activities but both type I and type III IFNs shared the same pathways pertinent to the growth inhibition.

The antiproliferative activity of type III IFNs was demonstrated in a certain type of tumors [25–27] and in nontumorous intestinal epithelial cells [17]. The scope of type III IFNs sensitivity is primarily dependent on the receptor expression as well as cell-type specificity as mentioned. The antiproliferative action, when more potent to tumors than to the normal counterparts, can be beneficial for cancer treatments. It is however relatively difficult to compare such preferential sensitivity with paired cell lines, normal and tumorous cells of the same cell origin. In esophagus, Het-1A, a nontumorous cell line immortalized with SV40 T antigen, is completely resistant to the type III IFNs-mediated growth inhibition despite being positive for the receptors. Some esophageal carcinoma cells however were also insensitive, and the preferential tumor susceptibility remains unknown in esophageal carcinoma. In addition, both intestinal epithelial cells and colon carcinoma cells were susceptible to type III IFNs [17]. Although no comparative data between tumors and nontumorous cells were available, the preferential inhibitory action to tumors may not be well evidenced. On the other hand, type I IFNs may have such propensity to induce cell death in tumors rather than nontumorous cells. The preferential inhibition could be linked with better proliferative activity of tumors compared with the normal counterparts but no conclusive data are currently available as to the preferential cytotoxicity to tumors with type I and type III IFNs.

The growth inhibition with type I and type III IFNs was directly evidenced by the decreased cell numbers as well as colorimetric assays. The activity is linked with tyrosine phosphorylation of IL-28R*α* at residues of 343 and 517, which leads to optimal activation of STAT2 [28]. We recently demonstrated 2 modes of the growth inhibition, cell cycle arrest at G1-phase and apoptosis [24]. The cell cycle stop was accompanied by augmented p21 expression and pRb dephosphorylation, which seem to be independent on p53 signaling pathways. The same biochemical changes were also demonstrated with murine tumor cells [27]. IFN-*λ*1 induced cell death in some of esophageal carcinoma cells by activating sequential caspase cleavage cascades including both intrinsic and extrinsic apoptotic pathways. We initially thought that IFN-*λ*1 induced G1 arrest and subsequently apoptosis but this was not the case. The choice of either G1 arrest or sub-G1 induction was dependent on the cell types. As mentioned, induction of G1-phase arrest or increased sub-G1 fractions by apoptosis was not observed in all the carcinoma cells tested. It is also interesting whether G1-arrested cells with type III IFNs were subjected to the same G1 arrest with type I IFNs. We found that type I IFNs did not induced such G1 arrest in the cells, suggesting a possible discrete pathways between type I and type-III-IFNs-mediated signaling. These studies suggested that the same cell repertoire within the identical lineage was susceptible to both type I and type III IFNs in the growth inhibitory action but the mechanisms were dependent on the cell type specificity.

## 5. Effects on Immune Systems

Type I IFNs have a wide range of immune stimulatory activities, but the main action is to augment T helper type 1 (Th1) cell responses, enhancing expression of MHC class I molecules and generating natural-killer- (NK-) cell- and T-cell-mediated cytotoxicity. Type I IFNs thus function to elevate both innate and adaptive immune responses. Type III IFNs seem to support cell-mediated immunity by upregulating the class I expression, but there has not been enough evidence to demonstrate that type III IFNs activate directly immune cells and induce production of Th1 cytokines. A recent study showed that IFN-*λ*1 diminished IL-13 levels and elevated IFN-*γ* production; however, subsequent study suggested that peripheral blood cells treated with IFN-*λ*1 rather upregulated expression levels of IL-6, IL-8, and IL-10 but not TNF-*α* or IL-1*β*, suggesting the role in Th2 differentiation but not in inflammatory reactions [29]. IFN-*λ*1 however has not been demonstrated to increase antibody formation despite augmented Th2 cytokine production. In addition, IFN-*λ*1 also elevated transcription of the *monokine induced by IFN-*γ**(*Mig*) and the *IFN-*γ* inducible protein-10* (*IP-10*) genes and in peripheral blood cells [30]. These molecules favor for antiangiogenesis, which consequently suppress tumor growth. These data collectively imply that type III IFNs have similar immune regulatory activities as type I IFNs but could have some distinct properties. Moreover, type III IFNs activate STAT4 molecules which are not stimulated by type I IFNs through their phosphorylation, implying that type-III-IFN-mediated effects to immune systems are not identical to those with type I IFNs. Interestingly, Mennechet and Uzé reported contradictory data that type-III-IFNs-treated dendritic cells induced FOXP3-positive regulatory T cells [14] although meticulous further studies are required regarding the immune tolerance or the suppressive factions.

In* in vivo* settings, secretion of type III IFNs from tumors achieved antitumor responses against the transduced tumors. Numasaki et al. showed that local secretion of mIFN-*λ*2 from murine tumors produced antitumors responses which were mediated by neutrophils, NK and CD8-positive T cells [31]. The study also showed that IFN-*γ* but not IL-12, IL-17, or IL-23 was essential for the antitumor responses. Sato et al. demonstrated that NK and perhaps NKT cells played a crucial role in the antitumor effects with less significant involvement of cytotoxic T cells [27] although an *in vitro *assay showed that type III IFNs did not augment NK activities [13]. These results were inconsistent but suggest that type III IFNs induce immune responses, initially innate and sequentially adaptive immunity against tumors. In contrast, Lasfar et al. demonstrated intriguing results with murine B16 melanoma expressing mIFN-*λ*2 [7]. The growth of the transduced tumors was retarded, and even loss of the tumorigenicity was observed; however, mice that rejected the B16 tumors secreting mIFN-*λ*2 failed to induce immunological memory responses, suggesting that mIFN-*λ*2 does not contribute to adaptive immune responses. Moreover, they did not notice enhanced NK activities. These data suggest a possible mechanism by upregulated Mig and IP-10, both of which suppress neoangiogenesis within tumors. The mechanisms of cytotoxicity operating *in vivo* are different from that in *in vitro* studies, and several reasons besides the direct growth inhibitory action can explain the antitumor effects by type III IFNs, augmentation of MHC class I antigens expression which subsequently enhances antigenicity of tumors, a possible induction of Th1 type cytokines which increases antigen presenting and favors generation of cytotoxic T cells, and antiangiogenesis. These actions are also shared with type I IFNs and thereby specific immunological significance of type III IFNs remains unknown.

## 6. Conclusions and Prospects

Type III IFNs have multiple functions including antiviral, immunomodulatory, and antiproliferative actions, and the majority of the actions overlap with those of type I IFNs. A restricted expression of the type III receptor complex in contrast with an ubiquitous expression of type I IFNs receptors however suggests differential functions of the type III IFNs in *in vivo* settings. Several studies in fact demonstrated that differential activities between two types of IFNs in certain experimental models such as responsiveness to viral infections. Antitumor effects produced by type III IFNs may however not be different from those by type I IFNs except the tissues-dependent receptor distributions.

Feasible clinical applications of type III IFNs are determined by a number of factors including the biological activity and the potency. The antitumor effects *in vivo *of type III IFNs in comparison with type I IFNs are not well established, but the activities of type III IFNs seem to be less potent than those of type I IFNs from the standpoint of the MHC class I upregulation and the antiproliferative action although contradictory results were reported [19]. The restricted expression of type III IFNs receptors however can be a clue for the clinical application in term of cell-mediated delivery of type III IFNs to target tumors. Fibroblasts or endothelial cells, negative for type III IFNs receptors, are resistant to the IFNs-mediated apoptosis but can deliver the IFNs to the target cells in the vicinity. Transduction of such carrier cells with the *IFN* genes and injection of the cells into type-III-IFNs-sensitive target tumors can generate antitumor effects by inducing apoptotic cell death. Cell-mediated delivery of a soluble factor can be more beneficial than systemic administrations since local concentrations of the factor are relatively maintained in the delivery system. Continuous secretion of factors from the producing cells can produce better therapeutic effects and circumvent any possible adverse effects.

Recombinant type I IFNs have been tested for the antitumor effects against a variety of tumors in clinical settings. The clinical studies however did not reveal any significant benefits partly due to the toxicity in systemic administrations. Any combinatory use with several types of anticancer agents did not increase the effects in most of the trials [32]. Type III IFNs have not yet been investigated for the clinical efficacy, but the similarity of its intracellular signal pathways with type I IFNs, despite several advantages of type III IFNs, implies that type III IFNs may not be dramatically better as an anticancer agent than type I IFNs. We probably need a novel strategy to obtain clinical benefits with type III as well as type I IFNs, which includes a local administration in the form of encapsulated protein particles and perhaps viral and nonviral expression vector systems. Much of preclinical studies and clinical trials with such a novel delivery system will be a subject in future.

## Abbreviations

IFN: Interferon
STAT: Signal transducer and activator of transcription
IL: Interleukin-29
TLRs: Toll-like receptors
RIG-1: Retinoic acid inducible gene-I
JAK: Janus tyrosine kinase
TYK2: Tyrosine kinase 2
ISREs: IFN-stimulated response elements
ISGs: IFN-stimulated genes
IRF-9: IFN-regulatory factor-9
GAS: IFN-*γ* activation site
MxA: Myxovirus resistance protein
MHC: Major histocompatibility complex
PI3K: Phosphatidyl-inositol-3-kinase
MAP: Mitogen-activated protein
Th1: T helper type 1
NK: Natural killer
Mig: Monokine induced by IFN-*γ*
IP-10: IFN-*γ* inducible protein-10.
